# Role of Preoperative Neutrophil-Lymphocyte Ratio in Predicting Prognosis After Liver Transplantation for Chronic Liver Failure

**DOI:** 10.7759/cureus.80749

**Published:** 2025-03-18

**Authors:** Surbhi Abrol, Manish Tandon, Arun M Raghu, Chandrakant Pandey

**Affiliations:** 1 Department of Anesthesia and Critical Care, Northampton General Hospital, Northampton, GBR; 2 Department of Anesthesiology, Dharamshila Narayana Superspeciality Hospital, Delhi, IND; 3 Department of Anesthesiology and Transplant Anesthesiology, Gleneagles Global Hospital, Bangalore, IND; 4 Department of Anesthesiology, Medanta Hospital, Lucknow, IND

**Keywords:** anaesthesiology, chronic liver disease, critical care, hepatology, mortality, neutrophil lymphocyte ratio, transplant

## Abstract

Introduction: The neutrophil-lymphocyte ratio (NLR) is an easily calculable biomarker known to have a predictive value in cardiac disease, malignancy, and renal failure. However, it has not been studied before in chronic liver disease patients undergoing liver transplantation. We aimed to evaluate the role of the pre-transplantation NLR in predicting the prognosis of patients with chronic liver failure undergoing liver transplantation.

Method: Data was retrospectively collected from 46 patients with chronic liver disease who underwent liver transplantation. The patients were divided into two groups. Group A had 23 patients who survived after liver transplantation. Group B had 23 patients who did not survive. NLR was calculated by dividing the percentage of neutrophils by the percentage of lymphocytes in peripheral blood. The NLR cut-off value was based on a receiver operating characteristic curve analysis. Postoperative complications were also noted.

Results: Preoperative NLR of 3.46 can predict post-transplantation mortality, with the area under the curve (AUC) of 0.86, having a sensitivity of 86.96% and a specificity of 73.91%. NLR emerged as an independent predictor of mortality (hazard ratio (HR) = 4.1, p = 0.028) after adjusting for the Model for End-Stage Liver Disease-Sodium (MELD-Na), creatinine, and neutrophil count. A rising NLR trend was significantly associated with the development of postoperative complications like neurological disease (p < 0.001), coagulopathy (p = 0.004), and acute kidney injury (p = 0.043).

Conclusion: A high preoperative NLR is a predictor of poor outcomes in liver transplantation patients with chronic liver disease.

## Introduction

Liver transplantation is the definitive treatment for end-stage liver disease, yet predicting post-transplantation outcomes remains a challenge [1]. Pathophysiology of liver disease, irrespective of the cause, includes inflammation and necrosis. Neutrophils play a crucial role in this inflammatory process, contributing to hepatic tissue damage in hepatitis B virus (HBV) and hepatitis C virus (HCV) infections, as well as in non-alcoholic steatohepatitis (NASH). In alcoholic liver disease, neutrophil migration from the sinusoids into the liver parenchyma is a critical step in hepatic inflammation [2].

Many mechanisms are described to explain the occurrence of lymphopenia seen in chronic liver disease. Lymphopenia is a clinical feature of hepatitis-associated aplastic anemia (HAAA), resulting from severe bone marrow failure caused by hepatitis B and C viruses [3]. In cirrhosis, impaired production of new T lymphocytes due to increased aging and atrophy of the thymus can result in lymphopenia. Additionally, lymphopenia is known to be an outcome of splenic sequestration and consumption of T lymphocytes related to activation-driven bacterial translocation and increased apoptosis. It is also linked to their impeded compensatory proliferation [4].

Neutrophil-lymphocyte ratio (NLR) is an index of inflammation that can be easily derived from peripheral blood count. This marker of systemic inflammation shows the relationship between two distinct immune pathways. Neutrophil count reflects an ongoing disruptive inflammation, whereas lymphocyte count portrays the healing immune regulatory process. High NLR has been reported in patients with NASH and advanced fibrosis [5]. NLR has also been shown to predict mortality in cirrhosis of the liver independent of Child-Turcotte-Pugh (CTP) and Model for End-Stage Liver Disease (MELD) scores. More importantly, NLR can predict mortality even in patients with low MELD scores [6]. It has been extensively demonstrated by numerous studies that high NLR can predict poor outcomes in conditions like cardiac disease, malignancy, and renal failure [7-10].

Several other inflammatory indicators, including systemic inflammatory response syndrome (SIRS), C-reactive protein (CRP), and MELD scores, have prognostic value in liver disease but also have limitations. SIRS, irrespective of the presence of infection, is shown to have a significant prognostic value in patients with cirrhosis and acute renal function failure [11]. It has been linked with high grades of hepatic encephalopathy in cirrhosis [12]. Moreover, it occurs frequently in advanced cirrhosis and leads to high in-hospital mortality [13]. However, the clinical indicators of SIRS can be potentially masked by the clinical signs and symptoms of cirrhosis; hence, the degree of the systemic inflammatory response mounted by these patients does not accurately reflect their development of SIRS [14]. A persistently elevated level of CRP, a biomarker of inflammation, is shown to be better than SIRS in predicting short-term mortality in cirrhosis. However, CRP is synthesized by the liver and is seen to be elevated only slightly even in patients with an active infection [15]. Furthermore, various factors such as body mass index, weight loss, cigarette smoking, high blood pressure, alcohol consumption, and diabetes can affect the levels of CRP [16]. MELD score, widely utilized for the allocation of chronic liver disease patients for liver transplantation, is also considered to be a poor predictor of survival after liver transplantation surgery [17].

A need for an accurate prognostic predictor of post-liver transplantation outcomes in patients with chronic liver disease has long been felt. It has become pertinent to obtain a reliable prognostic marker, given the high costs and scarcity of resources, both human and material, for optimum utilization of the resources. NLR is an inexpensive, non-invasive, and easily calculable value that can be obtained from peripheral blood count. In this study, we aimed to analyze the role of NLR in predicting mortality and morbidity in patients with chronic liver disease undergoing liver transplantation.

## Materials and methods

A hazard ratio (HR) of 4.305 has been reported in acute-on-chronic liver failure patients [18]. Considering a 50% reduction in this HR following liver transplantation, we needed to study 30 patients to achieve an outcome with a power of 80% and a 5% level of two-tailed significance.

After obtaining approval from the Institutional Review Board of the Institute of Liver and Biliary Sciences, New Delhi, this single-center study was designed. The informed consent requirement was waived (F/25/5/81/ILBS/AC/2015/1302-04; dated 23/04/2016). This study was conducted in 2016 at the Institute of Liver and Biliary Sciences, New Delhi, India. This retrospective matched case-control study was conducted on 46 patients divided into two groups: group A comprised 23 patients who survived after liver transplantation and group B comprised 23 patients who died after liver transplantation. We included patients undergoing liver transplantation for chronic liver disease of any etiology, aged 18-70 years, with a BMI of 18-30 and an MELD score of ≥15. The eligibility criteria for liver transplantation followed the American Association for the Study of Liver Diseases (AASLD) practice guidelines. The operative methods for liver transplantation included both dead-donor-liver transplantation (DDLT) and live-donor-liver transplantation (LDLT). To minimize the confounding by infection in our analysis, we excluded patients with known foci of infection, positive microbiological culture, acute liver failure, or acute-on-chronic-liver failure (ACLF). Additionally, patients with hepatocellular carcinoma (HCC), which is not a known cause of cirrhosis, and those with a history of steroid and/or immunosuppressant administration that could influence the NLR and ABO-incompatible liver transplantation patients were also excluded.

We performed a retrospective review of clinical charts, operation records, and electronic data of 46 patients who had undergone liver transplantation at our hospital between January 2010 and December 2015.

Preoperative investigations done one day before the day of the surgery were taken as the baseline investigations. Data from the patient’s first postoperative day until the day of discharge from the hospital or until mortality was collected. Data from postoperative investigations done as a part of the institution's postoperative protocol were used for this study. NLR was calculated by dividing the percentage of neutrophils by the percentage of lymphocytes in peripheral blood. For the purpose of comparison with the normal population, an NLR of 2.15 was considered normal [19].

Complications were also noted. Coagulopathy was defined by the presence of at least one of the following criteria: activated partial thromboplastin time (aPTT) > 38 seconds, international normalized ratio (INR) > 1.4, or platelet count < 80,000/cu mm [20]. Cerebrovascular accidents were confirmed on neuroimaging. Patients with postoperative seizures, which responded to tacrolimus withdrawal, were excluded from the data on neurological complications. For the diagnosis of acute kidney injury (AKI), we followed the Kidney Disease: Improving Global Outcomes (KDIGO) definition, which states that AKI is diagnosed by an absolute increase in serum creatinine, at least 0.3 mg/dL (26.5 μmol/L) within 48 hours, or by a 50% increase in serum creatinine from baseline within seven days, or a urine volume of less than 0.5 mL/kg/h for at least six hours [21]. For this analysis, we excluded patients with known AKI prior to the surgery.

A histidine-tryptophan-ketoglutarate (HTK) solution was used for organ preservation. As a part of the post-transplantation-immunosuppression protocol of our institute, all patients received methylprednisolone 100 mg in the anhepatic phase, which was then tapered over five days. Tacrolimus, mycophenolate mofetil, and prednisolone were given as maintenance immunosuppressants. Antimicrobial prophylaxis included piperacillin-tazobactam, teicoplanin, metronidazole, and fluconazole, administered an hour before the surgery and continued 72 hours post-transplantation. Additionally, patients received a 20% albumin infusion until serum albumin levels exceeded 3 g/dL. All procedures performed in this study complied with the Declaration of Helsinki.

All results and observations obtained were thoroughly analyzed as per standard statistical methods. Data was reported as proportions or mean ± SD. The chi-square test, or Fisher exact test, was used for categorical variables. Normally distributed continuous variables were compared using the Student t-test (unpaired data) and paired t-test (paired data) to analyze the significant effect of NLR between pre- and post-liver transplantation. One-way analysis of variance (ANOVA) was used to compare preoperative NLR among different liver disease etiologies. The Mann-Whitney U test (unpaired data) or the Wilcoxon Signed Rank test (paired data) was used for non-parametric data. The area under the curve was calculated using the receiver operating characteristic (ROC) curve method. Cox proportional hazards regression analysis was used to evaluate the association between the NLR cut-off and post-transplantation survival. Binary logistic regression analysis was performed to identify independent predictors of mortality. Variables with a p-value < 0.2 in univariate analysis were included in the multivariate logistic regression model. The overall model fit was assessed using the Hosmer-Lemeshow goodness-of-fit test, and the proportion of variance explained by the model was evaluated using Nagelkerke R². IBM SPSS Statistics for Windows, Version 23 (Released 2015; IBM Corp., Armonk, New York, United States) was used for analysis. A p-value of less than 0.05 was considered statistically significant.

## Results

Sex ratios were comparable, as the survivor group had a sex ratio of 19:4 and the non-survivors had a sex ratio of 18:5, p = 1.00 (p > 0.05). The overall sex ratio was 37:9. The etiology of liver disease among patients in our study included alcoholic liver disease, cryptogenic cirrhosis, NASH, HCV cirrhosis, and HBV cirrhosis. The preoperative NLR distribution across these groups was similar with p = 0.329 (p > 0.05). Additionally, the groups in our study were comparable for the aforementioned liver disease etiologies, mean age, mean duration of surgery, mean cold ischemia time, mean warm ischemia time, mean graft-to-recipient weight ratio (GRWR), mean preoperative creatinine, mean preoperative bilirubin, mean preoperative MELD score, mean preoperative Model for End-Stage Liver Disease-Sodium (MELD-Na) score, mean preoperative Child-Turcotte Pugh (CTP) score, mean preoperative total leucocyte count (TLC), mean preoperative neutrophil count, and mean preoperative platelet-lymphocyte ratio (PLR) (p > 0.05). However, when we calculated the means for preoperative NLR in the survivors and the non-survivors, the difference was found to be significant (p < 0.05). This can be seen in Table 1.

**Table 1:** Comparison of preoperative values in two groups The chi-square test and t-test were applied wherever applicable; p < 0.05 was considered significant. Sex ratio: male-female ratio; age: in years; NASH: non-alcoholic steatohepatitis; HCV: hepatitis C virus; HBV: hepatitis B virus; mean duration of surgery: in hours; mean cold ischemia time: in minutes; mean warm ischemia time: in minutes; GRWR: graft-to-recipient weight ratio; mean preoperative creatinine: in mg/dL; mean preoperative bilirubin: in mg/dL; MELD: Model for End-Stage Liver Disease score; MELD-Na: Model for End-Stage Liver Disease-Sodium score; CTP: Child-Turcotte-Pugh score; TLC: total leucocyte count; PLR: platelet-lymphocyte ratio; NLR: neutrophil-lymphocyte ratio; χ²: chi-square statistic; (df): degrees of freedom

Parameter	Survivors	Non-survivors	χ² (df)/t-value	p-value
Sex ratio	19:4	18:5	1.267 (1)	1.00
Age	41.43 ± 5.17	47.43 ± 5.26	0.906	0.835
Alcoholic liver disease	9 (39.13%)	10 (43.48%)	1.595 (4)	0.329
Cryptogenic cirrhosis	4 (17.39%)	6 (26.09%)		
NASH	4 (17.39%)	3 (13.04%)		
HCV cirrhosis	3 (13.04%)	1 (4.35%)		
HBV cirrhosis	3 (13.04%)	3 (13.04%)		
Mean duration of surgery	15.80 ± 3.07	15.74 ± 2.54	0.080	0.937
Mean cold ischemia time	71.45 ± 14.89	38.92 ± 8.11	0.661	0.512
Mean warm ischemia time	51.91 ± 23.56	52.82 ± 13.03	0.163	0.872
Mean GRWR	1.0080 ± 0.2104	1.0503 ± 0.2608	0.605	0.548
Mean preoperative creatinine	0.7687 ± 0.357	0.8174 ± 0.329	1.371	0.177
Mean preoperative bilirubin	6.8122 ± 6.65	7.8548 ± 5.21	0.592	0.557
Mean preoperative MELD score	21.43 ± 5.51	23.61 ± 4.29	1.493	0.143
Mean preoperative MELD-Na score	23.74 ±5.86	26.61 ± 6.85	1.526	0.134
Mean preoperative CTP score	10.39 ± 1.88	11.04 ± 1.60	1.265	0.212
Mean preoperative TLC	6.331 ± 5.008	5.626 ± 2.690	0.595	0.555
Mean preoperative neutrophil	63.013 ± 20.941	70.043 ± 9.522	1.466	0.150
Mean preoperative PLR	5.244 ± 4.728	5.075 ± 4.016	0.131	0.897
Mean preoperative NLR	2.88 ± 1.77	6.62 ± 4.69	3.5	<0.001

**Figure 1:**
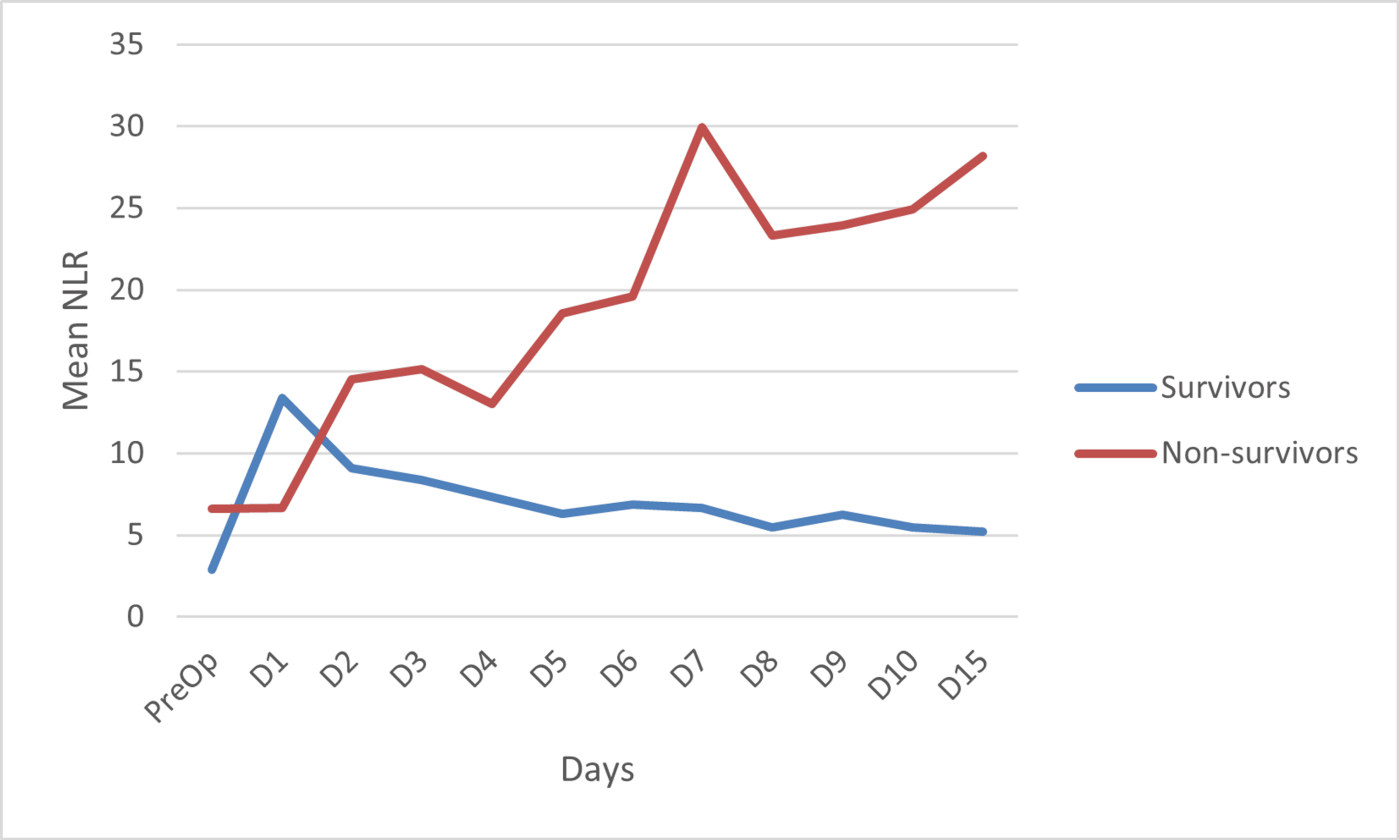
Trend of neutrophil-lymphocyte ratio (NLR) over time between the survivors and the non-survivors NLR: neutrophil-lymphocyte ratio; PreOp: preoperative; D: postoperative day

As depicted in Figure 1, on postoperative day 1, compared to their respective preoperative values, NLR in the survivors exhibited a steep rise. In contrast, in the non-survivors, it showed a marginal increase (from 6.62 to 6.66). From postoperative day 2 onward, NLR in the survivors began to decline, whereas in the non-survivors it did not decrease, following a rather upward trajectory until postoperative day 15.

The difference in postoperative NLR between the survivors and the non-survivors on each day was statistically significant, as evident from Table 2. 

**Table 2:** Neutrophil-lymphocyte ratios (NLR) in the survivors and the non-survivors on postoperative days Data are presented as mean ± standard deviation. A t-test was applied, and p < 0.05 was considered statistically significant NLR: neutrophil-lymphocyte ratio; POD: postoperative day

Postoperative NLR survivors versus non-survivors											
	POD 1	POD 2	POD 3	POD 4	POD 5	POD 6	POD 7	POD 8	POD 9	POD 10	POD 15
Survivors	13.409 ± 6.259	9.097 ± 5.474	8.383 ± 4.292	7.306 ± 3.851	6.268 ± 3.646	6.866 ± 3.200	6.670 ± 3.483	5.474 ± 2.241	5.457 ± 2.193	6.271 ± 3.236	5.536 ± 2.709
Non-survivors	6.664 ± 2.276	14.522 ± 4.813	15.118 ± 6.068	13.013 ± 5.637	18.554 ± 6.004	19.596 ± 7.021	22.936 ± 8.216	23.341 ± 9.106	24.747 ± 7.738	25.305 ± 6.065	28.167 ± 3.666
t-value	4.857	3.569	4.313	3.981	8.339	7.747	8.391	9.045	9.716	9.752	11.962
p-value	< 0.001	< 0.001	< 0.001	< 0.001	< 0.001	< 0.001	< 0.001	< 0.001	< 0.001	< 0.001	< 0.001

**Figure 2:**
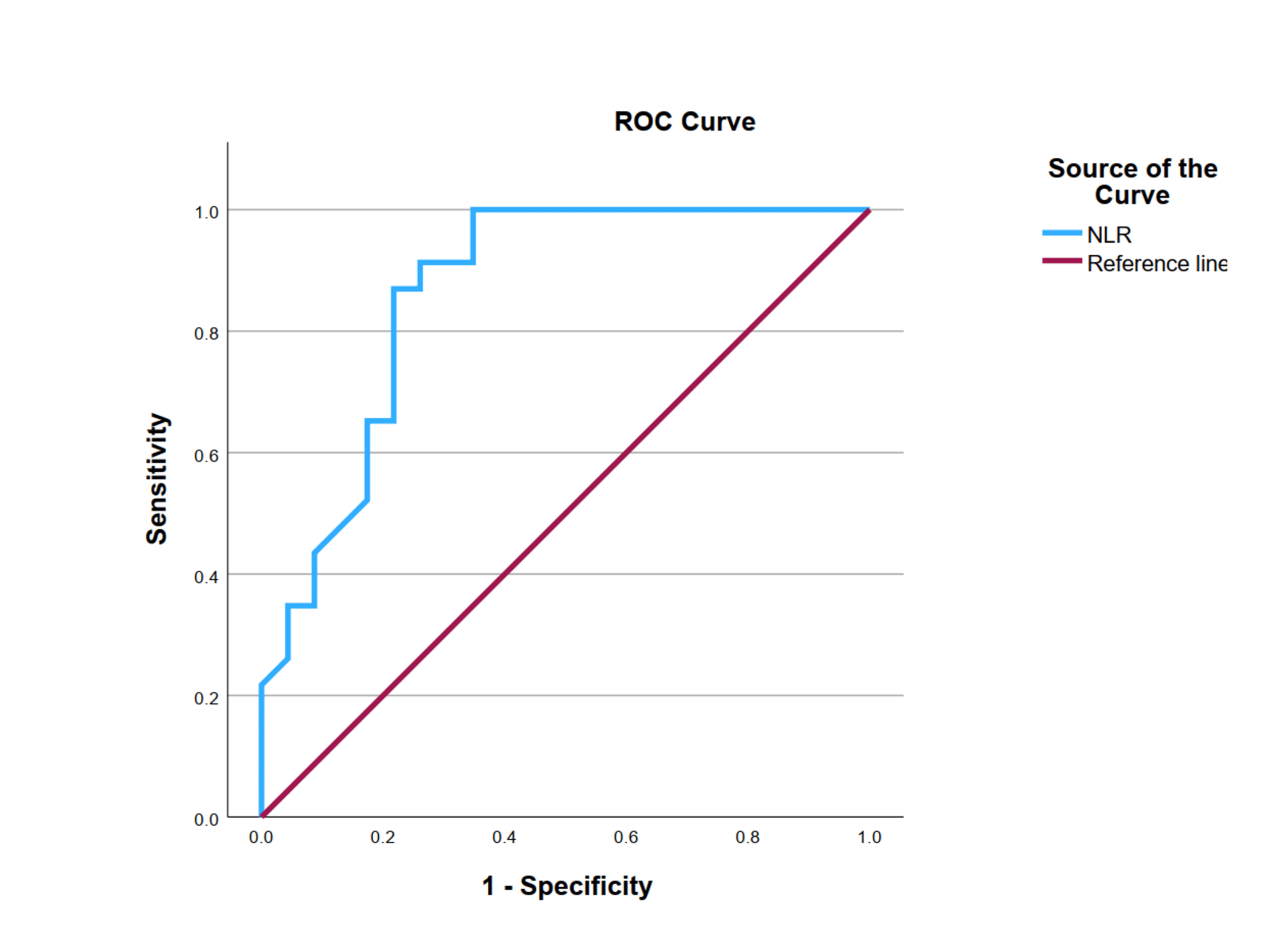
ROC curve of preoperative NLR ROC: receiver operating characteristic curve; NLR: neutrophil-lymphocyte ratio

When preoperative NLR was analyzed on the ROC curve, the area under the curve (AUC) was calculated to be 0.865 (95% CI: 0.757-0.973). This is shown in Figure 2.

Using the Youden Index (Jmax 0.652), an optimal cut-off of NLR was identified as 3.46. As summarized in Table 3, this has a sensitivity of 86.96% and a specificity of 73.91% for the prediction of mortality. The positive likelihood ratio was found to be 3.33, and the negative likelihood ratio was 0.18.

**Table 3:** Values for NLR cut-off of 3.46 NLR: neutrophil-lymphocyte ratio

Measure	Value	95% confidence interval
Sensitivity	86.96%	66.41% to 97.22%
Specificity	73.91%	51.59% to 89.77%
Positive likelihood ratio	3.33	1.65 to 6.75
Negative likelihood ratio	0.18	0.06 to 0.52

The Cox regression model suggested that a cut-off NLR of 3.46 is a significant predictor of mortality (HR = 4.096, 95% CI: 1.651-14.401, p = 0.028). Patients with NLR >3.46 in our study faced a 4.1 times greater hazard of post-transplantation mortality compared to those with NLR <3.46.

Univariate logistic regression identified NLR as a significant predictor of mortality (p < 0.05). Although preoperative MELD-Na score (p = 0.138), creatinine (p = 0.183), and neutrophil count (p = 0.167) were not statistically significant, their p-values between 0.1 and 0.2 justified their inclusion in the multivariate analysis alongside NLR to ensure a thorough evaluation.

In the multivariate logistic regression model, NLR emerged as the only independent predictor of mortality (p = 0.012; OR = 2.446, 95% CI: 1.122-4.903), indicating that patients with higher NLR had approximately 2.45 times the odds of mortality compared to those with lower NLR. MELD-Na (p = 0.870), creatinine (p = 0.230), and neutrophil count (p = 0.412) remained insignificant predictors. The overall logistic regression model was statistically significant (χ²(4) = 20.452, p < 0.001), explaining 47.9% of the variance in mortality (Nagelkerke R² = 0.479). The Hosmer-Lemeshow goodness-of-fit test indicated the model fit the data well (χ²(7) = 11.830, p = 0.106). The analysis is presented in Table 4.

**Table 4:** Univariate and multivariate analysis of factors affecting survival after liver transplantation NLR: neutrophil-lymphocyte ratio; MELD-Na: Model for End-Stage Liver Disease-Sodium score; CIT: cold ischemia time; WIT: warm ischemia time; GRWR: graft-to-recipient weight ratio; CTP: Child-Turcotte-Pugh score; MELD: Model for End-Stage Liver Disease score; TLC: total leucocyte count; PLR: platelet-lymphocyte ratio; OR: odds ratio

	Univariate analysis				Multivariate analysis			
Variable	p-value	OR	95% confidence interval		p-value	OR	95% confidence interval	
NLR	0.005	2.063	1.241	3.427	0.012	2.446	1.122	4.903
Creatinine	0.183	3.529	0.552	22.563	0.230	3.439	0.457	25.847
MELD-Na	0.138	1.076	0.977	1.186	0.870	0.989	0.871	1.124
Neutrophil	0.167	1.030	0.988	1.075	0.412	0.974	0.915	1.037
Duration of surgery	0.935	0.991	0.804	1.222				
CIT	0.510	1.004	0.993	1.015				
WIT	0.868	1.003	0.972	1.034				
GRWR	0.539	2.199	0.177	27.266				
CTP	0.209	1.247	0.884	1.759				
MELD	0.202	1.099	0.967	1.248				
Bilirubin	0.549	1.031	0.933	1.140				
TLC	0.551	0.954	0.818	1.113				
PLR	0.894	0.991	0.866	1.134				

ROC analysis was also done for preoperative values of total leukocyte count (TLC), neutrophils, lymphocytes, platelets, lymphocyte-monocyte ratio (LMR), and PLR. None of the AUC values demonstrated an acceptable discrimination to merit further analysis. This can be seen in Table 5.

**Table 5:** AUC for preoperative values of TLC, neutrophils, lymphocytes, platelets, LMR, and PLR AUC: area under the curve; TLC: total leucocyte count; LMR: lymphocyte-monocyte ratio; PLR: platelet-lymphocyte ratio

Preoperative values	AUC
TLC	0.485
Neutrophils	0.393
Lymphocyte	0.402
Platelets	0.509
LMR	0.522
PLR	0.517

We also evaluated NLR between the two groups of DDLT and LDLT on each day and found no significant difference between the two groups as shown in Table 6.

**Table 6:** NLR on each day in DDLT versus LDLT groups NLR: neutrophil-lymphocyte ratio; DDLT: dead donor liver transplantation; LDLT: live donor liver transplantation; Pre: preoperative; POD: postoperative day; Asym. Sig.: asymptotic significance; Exact Sig.: exact significance

DDLT vs. LDLT	NLR pre	NLR POD 1	NLR POD 2	NLR POD 3	NLR POD 4	NLR POD 5	NLR POD 6	NLR POD 7	NLR POD 8	NLR POD 9	NLR POD 10	NLR POD 15
Mann-Whitney U	85	79.5	69	73	66.5	85.5	93	73	87.5	67.5	89	29
Wilcoxon	140	289.5	279	283	276.5	295.5	148	128	142.5	112.5	144	50
Z	-0.609	-0.902	-1.365	-1.88	-1.474	-0.638	-0.308	-1.188	-0.55	-1.87	-0.276	-0.656
Asym. Sig (2 tailed)	0.509	0.367	0.172	0.235	0.14	0.523	0.758	0.235	0.582	0.061	0.783	0.512
Exact Sig. (2*(1-tailed Sig))	0.53	0.373	0.183	0.248	0.143	0.53	0.779	0.248	0.588	0.061	0.804	0.553

When compared with the mean NLR in a normal healthy population, which is reported to be 2.15 [19], the NLR in our patients on each day remained significantly elevated until postoperative day 15, as evident in Table 7.

**Table 7:** NLR on each day compared to the population mean of 2.15 NLR: neutrophil-lymphocyte ratio; pre: preoperative; POD: postoperative day; t: Student t-statistic; df: degrees of freedom

Population NLR = 2.15							
			Significance			95% confidence interval of the difference	
	t	df	One-sided p-value	Two-sided p-value	Mean difference	Lower	Upper
NLR pre	4.427	45	< 0.001	< 0.001	2.59935	1.4167	3.7820
NLR POD 1	9.621	45	< 0.001	< 0.001	10.49174	8.2954	12.6881
NLR POD 2	10.095	45	< 0.001	< 0.001	9.68087	7.7494	11.6123
NLR POD 3	10.443	44	< 0.001	< 0.001	9.76489	7.8805	11.6493
NLR POD 4	9.066	44	< 0.001	< 0.001	8.16022	6.3463	9.9742
NLR POD 5	8.018	44	< 0.001	< 0.001	6.46422	4.8394	8.0891
NLR POD 6	8.466	40	< 0.001	< 0.001	7.68512	5.8504	9.5199
NLR POD 7	8.298	36	< 0.001	< 0.001	6.84784	5.1741	8.5216
NLR POD 8	6.195	36	< 0.001	< 0.001	5.87865	3.9541	7.8032
NLR POD 9	6.431	35	< 0.001	< 0.001	6.73139	4.6065	8.8563
NLR POD 10	7.203	32	< 0.001	< 0.001	6.79606	4.8743	8.7178
NLR POD 15	5.623	18	< 0.001	< 0.001	7.09737	4.4454	9.7494

**Figure 3:**
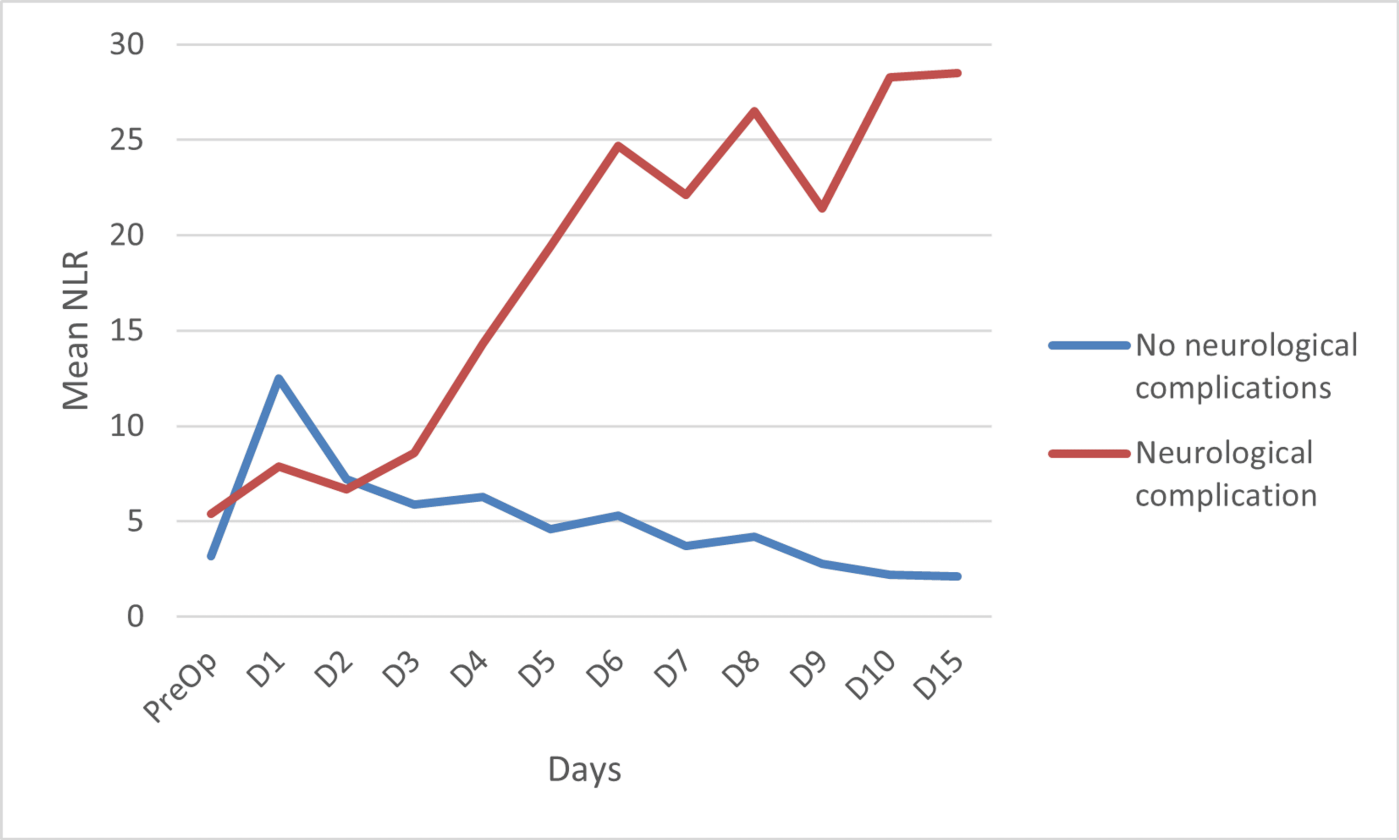
NLR on each day in patients with neurological complications compared to those who did not develop neurological complications NLR: neutrophil-lymphocyte ratio; PreOp: preoperative; D: postoperative day

A total of 12 patients (26%) in our study developed neurological complications like cerebrovascular accident (as evidenced by neuroimaging) and seizures (non-responsive to tacrolimus withdrawal). When NLR was analyzed on each day, the patients who had developed these complications showed a rising trend in NLR, whereas those without neurological complications showed a fall in NLR. The difference between the two groups was statistically significant with p < 0.001. This is depicted in Figure 3.

**Figure 4:**
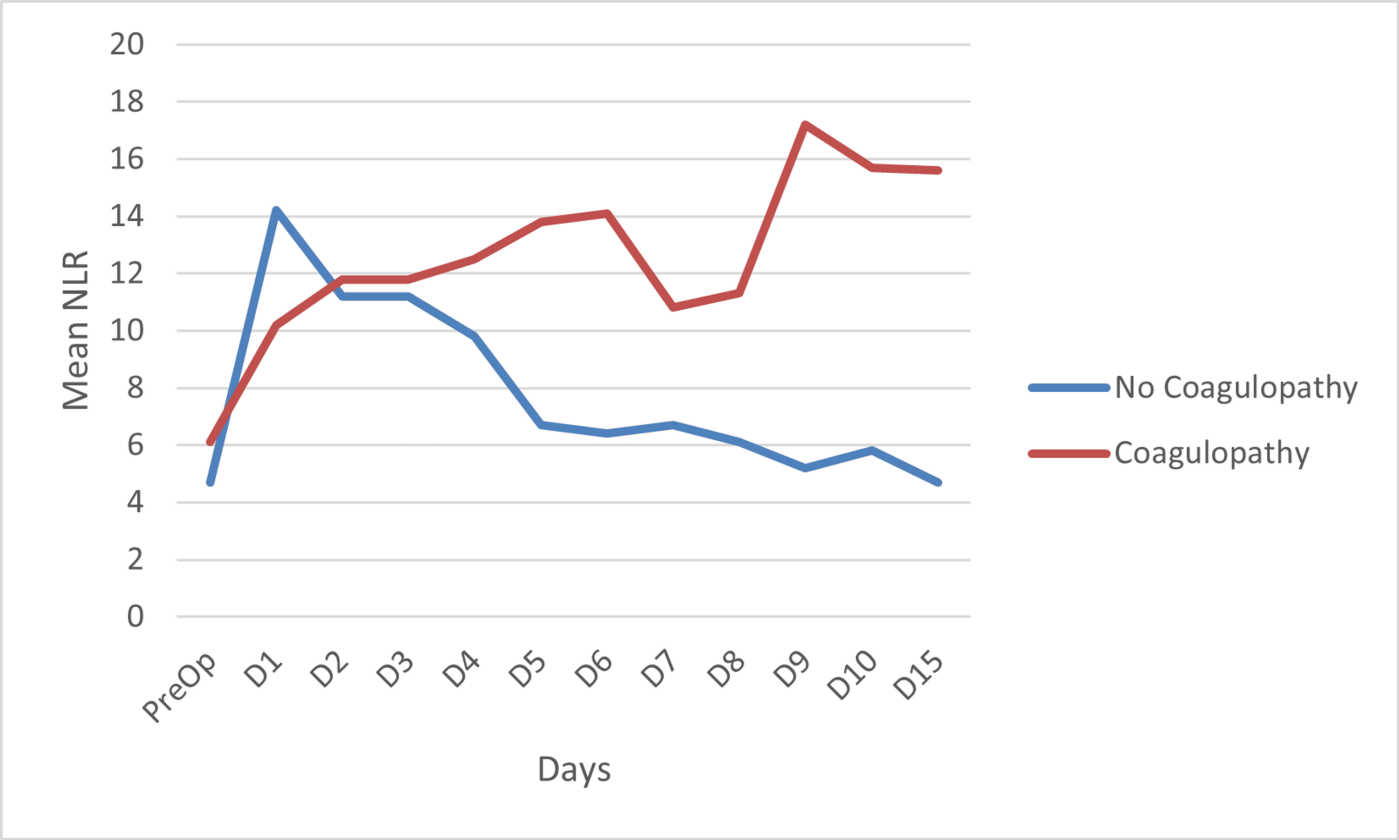
NLR on each day in patients with coagulopathy compared to those who did not develop coagulopathy NLR: neutrophil-lymphocyte ratio; PreOp: preoperative day; D: postoperative day

Fourteen patients (30.4%) in our study developed coagulopathy, which was also confirmed on a thromboelastogram (TEG). These patients presented with bleeding esophageal varices and/or melena, requiring blood product transfusion as guided by TEG. The patients with coagulopathy exhibited a rise in NLR values, whereas those who did not develop coagulopathy showed a fall in NLR values over the 15-day postoperative period. The difference between the two groups was statistically significant with p = 0.004 (p < 0.05). This is shown in Figure 4.

**Figure 5:**
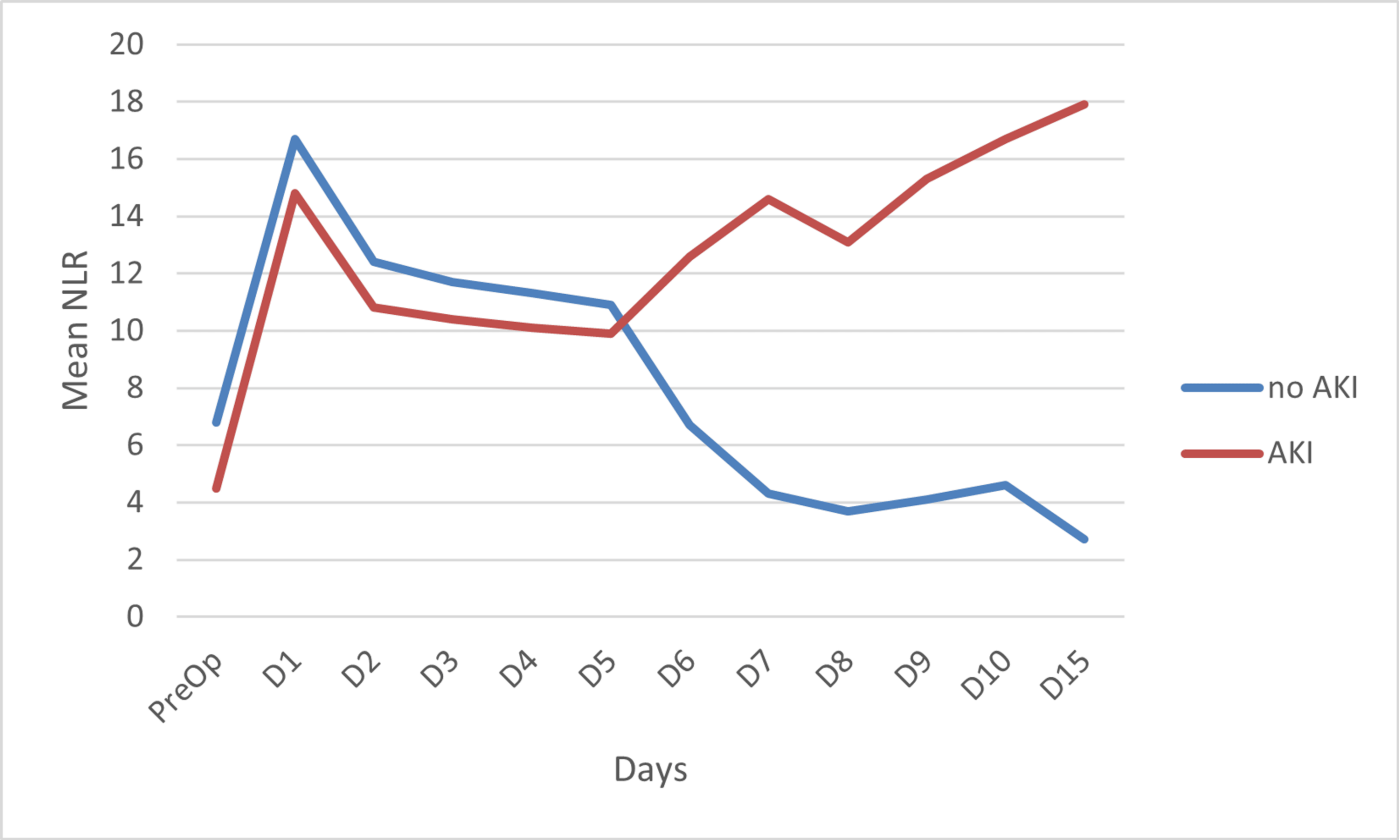
NLR on each day in patients with AKI compared to those who did not develop AKI NLR: neutrophil-lymphocyte ratio; AKI: acute kidney injury; PreOp: preoperative day; D: postoperative day

Fifteen patients (32.6%) were found to have a newly developed AKI during the study period. All of these patients required hemodialysis in the postoperative period. NLR followed a similar trend in both the AKI and the non-AKI patient groups until postoperative day 5. In both groups, NLR increased from preoperative day to postoperative day 1; this was followed by a decline until day 5. However, after day 5, NLR began to rise in AKI patients, whereas in the patients without AKI, NLR plummeted. The difference was statistically significant with p = 0.043 (p < 0.05). This is illustrated in Figure 5.

## Discussion

The findings of our study show that a high preoperative NLR can independently predict postoperative mortality and that a rising NLR trend is associated with complications in chronic liver disease patients undergoing liver transplantation. To the best of our knowledge, this is the first study to evaluate NLR in this group of patients.

There is evidence to link cirrhosis with SIRS. In a multicentric study by Thabut et al., SIRS emerged as a major independent prognostic factor in patients with cirrhosis and acute renal functional failure [11]. A study conducted by Cazzaniga et al. examined SIRS in 141 cirrhotic patients and reported that SIRS was significantly associated with the occurrence of portal hypertension and mortality in these patients [13]. Later, a study by Behroozian et al. observed that SIRS is a frequent occurrence in cirrhosis, it is associated with disease severity, and it adversely affects in-hospital outcomes [22]. Assessing the signs and symptoms of SIRS in cirrhotic patients can be challenging for several reasons. Hyperdynamic circulation commonly observed in cirrhosis, regardless of infection, often leads to tachycardia. Hypersplenism of cirrhosis reduces white blood cell counts, while hyperventilation occurs frequently in encephalopathy. Additionally, beta-blockers, widely prescribed for patients with cirrhosis, lower the heart rate [14,23]. Hence, there is a need for a reliable clinical marker to predict SIRS in this group of patients.

In cirrhosis, the basal level of CRP is found to be higher than that in non-cirrhosis patients. However, we chose not to evaluate CRP, as Pieri et al. observed in their study that in cirrhotic patients with infections, more severe liver dysfunction was associated with a lower increase in CRP [24]. Additionally, there is no agreed consensus on the cut-off value of CRP to define the severity of disease, and the requirement of two CRP measurements taken 15 days apart limits its use in the evaluation of disease severity in organ transplantation [14]. Moreover, numerous factors are known to influence CRP levels, including body mass index, weight loss, smoking, alcohol consumption, and diabetes [16].

Lymphocytopenia is more prevalent in cirrhosis than neutropenia [6]. Hepatitis viruses are reported to induce lymphopenia by suppressing the bone marrow [3]. Furthermore, thymic atrophy in cirrhosis impairs the production of new T lymphocytes. Splenic sequestration and increased cell consumption of lymphocytes induced by activation-driven bacterial translocation and an increase in apoptosis also contribute to lymphopenia in cirrhosis [4]. NLR, a surrogate biomarker of inflammation, has been shown to be a predictor of mortality in liver cirrhosis, even in patients with low MELD scores, as evidenced in a study by Biyik et al. [6]. In their study, Moreau et al. found that NLR was associated with mortality in critically ill cirrhotic patients with acute complications [25]. Earlier, Alkhouri et al. had reported in their patients who underwent liver biopsy that for each one-unit increase in neutrophil/lymphocyte (N/L) ratio, the likelihood of having NASH increased by 70%, and the likelihood of having liver fibrosis increased by 50%. The authors concluded that NLR could serve as a novel non-invasive marker to predict advanced disease in cirrhosis [5]. In light of the findings of the aforementioned studies, a study on NLR in patients with cirrhosis undergoing liver transplantation was warranted. NLR offers an advantage over other markers as it is cheap and easy to calculate, and its sampling, which is done as a part of routine investigations in transplantation patients, is easy to perform.

In our study, we observed an increase in NLR on the first postoperative day in both the survivors and the non-survivors compared to their respective preoperative values. We think this increase was likely attributed to the physiological stress response of the surgery. In the survivors, NLR began to decline from the second postoperative day, following an overall downward trajectory. In sheer contrast, NLR in the non-survivors increased slightly on postoperative day 1; thereafter, it exhibited a steep rise until postoperative day 8. Postoperative day 8 onwards, NLR rose even more sharply, exhibiting an overall increasing trend. The difference between the NLR of the survivors and the non-survivors was statistically significant. We believe the decrease in NLR in the survivors was likely due to the resolution of inflammation associated with chronic liver disease, which is known to harbor inflammation and SIRS. On the first postoperative day, NLR was found to be higher in the survivors than in the non-survivors. This finding was contrary to our expectations. We think the reason for this occurrence was possibly because of a more robust immune response mounted by the survivors compared to that of the non-survivors.

We also plotted an ROC curve and identified a preoperative NLR cut-off value of 3.46 having a sensitivity of 86.96% and a specificity of 73.91% to predict mortality. Lin et al. previously studied NLR in 150 ACLD patients who underwent liver transplantation [18]. They calculated a preoperative cut-off NLR of 4.16 to be predictive of mortality in their group of patients. Our cut-off NLR (3.46), in comparison, was found to be lower than theirs. We postulate that this difference may be due to distinct underlying pathological mechanisms - specifically, the presence of an acute event like acute-on-chronic liver failure in their cohort - which likely resulted in a higher NLR value compared to chronic liver disease pathology in our patients. Additionally, our smaller sample size may have contributed to the differences observed in NLR.

Our study also revealed that individuals with a higher NLR had a 4.1 times greater hazard of mortality compared to those with a lower NLR. Furthermore, NLR emerged as the only independent predictor of mortality in the multivariate model after adjusting for MELD-Na, creatinine, and neutrophil count (p = 0.012; OR = 2.446, 95% CI: 1.122-4.903). For each one-unit increase in NLR, the odds of mortality increased by approximately 145% compared to the previous unit. The model explained 47.9% of the variability in mortality, indicating a moderate predictive capability. Our findings are in line with those of Lin et al., who found that serum creatinine, in addition to NLR, was predictive of mortality, although the authors did acknowledge that the sensitivity of creatinine in their study was low [18].

Azab et al. reported a mean NLR of 2.15 in a cohort of 9427 healthy subjects [19]. For our study, we used this reference value to compare with the NLR in our patients on each day. We found a statistically significant difference between the two. This implies that chronic liver disease patients in our study had a higher NLR than in the normal population, likely due to underlying inflammation, and this inflammatory process appeared to continue up to day 15 after liver transplantation.

Limitations

There were some limitations to our study. It was a retrospective, single-center study with a relatively small sample size. This comes with an inherent restriction on generalizing the findings to broader populations. The role of NLR in predicting complications should be interpreted with caution, as our study was not adequately powered for this analysis.

All the patients in the survivor's group were discharged from the hospital at 15 days. Consequently, the Cox proportional hazards regression model may have been influenced by this early censoring. In light of this limitation, the analysis in our study primarily reflects short-term post-transplant outcomes, and caution should be exercised when extrapolating these results to long-term survival.

The post-transplant immunological milieu of the liver is influenced by various factors, including physiological surgical stress, the liver regenerative process, graft rejection, the presence of infection, the use of antibiotics, and the nutritional status of the patient. The complex interplay of these factors could potentially influence the ability of NLR to predict outcomes in our patients.

To eliminate the effect of immunosuppression, we excluded patients on immunosuppressants preoperatively from our study. Preoperative NLR values in our study were therefore not affected by the immunosuppression. However, unless contraindicated, all our patients receive immunosuppressants in the postoperative period following liver transplantation, as per our institution's protocol. To date, no studies have examined the effect of immunosuppressants on NLR in post-liver transplantation patients. Hence, we could not entirely eliminate the potential effect of postoperative immunosuppressants on NLR in our patients. This perhaps could be a subject of future studies.

## Conclusions

To sum up, our study demonstrates that preoperative NLR is a reliable predictive marker of mortality in chronic liver disease patients undergoing liver transplantation. Additionally, a rising NLR trend can serve as an early predictor of postoperative complications in this group of patients. NLR, a simple, rapid, and inexpensive marker, could be utilized for the optimal allocation of resources preoperatively and aid in the prediction and prevention of complications postoperatively. NLR could be the subject of larger studies and potentially find its place as a valuable adjunct in the future when scoring systems are made for the prediction of mortality and morbidity in these patients.

## References

[1] Zarrinpar A, Busuttil RW (2013). Liver transplantation: past, present and future. Nat Rev Gastroenterol Hepatol.

[2] Xu R, Huang H, Zhang Z, Wang FS (2014). The role of neutrophils in the development of liver diseases. Cell Mol Immunol.

[3] Rauff B, Idrees M, Shah SA (2011). Hepatitis associated aplastic anemia: a review. Virol J.

[4] Albillos A, Lario M, Álvarez-Mon M (2014). Cirrhosis-associated immune dysfunction: distinctive features and clinical relevance. J Hepatol.

[5] Alkhouri N, Morris-Stiff G, Campbell C (2012). Neutrophil to lymphocyte ratio: a new marker for predicting steatohepatitis and fibrosis in patients with nonalcoholic fatty liver disease. Liver Int.

[6] Biyik M, Ucar R, Solak Y (2013). Blood neutrophil-to-lymphocyte ratio independently predicts survival in patients with liver cirrhosis. Eur J Gastroenterol Hepatol.

[7] Bhat T, Teli S, Rijal J (2013). Neutrophil to lymphocyte ratio and cardiovascular diseases: a review. Expert Rev Cardiovasc Ther.

[8] Absenger G, Szkandera J, Pichler M (2013). A derived neutrophil to lymphocyte ratio predicts clinical outcome in stage II and III colon cancer patients. Br J Cancer.

[9] Sarraf KM, Belcher E, Raevsky E, Nicholson AG, Goldstraw P, Lim E (2009). Neutrophil/lymphocyte ratio and its association with survival after complete resection in non-small cell lung cancer. J Thorac Cardiovasc Surg.

[10] Reddan DN, Klassen PS, Szczech LA, Coladonato JA, O'Shea S, Owen WF Jr, Lowrie EG (2003). White blood cells as a novel mortality predictor in haemodialysis patients. Nephrol Dial Transplant.

[11] Thabut D, Massard J, Gangloff A (2007). Model for end-stage liver disease score and systemic inflammatory response are major prognostic factors in patients with cirrhosis and acute functional renal failure. Hepatology.

[12] Shawcross DL, Sharifi Y, Canavan JB (2011). Infection and systemic inflammation, not ammonia, are associated with Grade 3/4 hepatic encephalopathy, but not mortality in cirrhosis. J Hepatol.

[13] Cazzaniga M, Dionigi E, Gobbo G, Fioretti A, Monti V, Salerno F (2009). The systemic inflammatory response syndrome in cirrhotic patients: relationship with their in-hospital outcome. J Hepatol.

[14] Dirchwolf M, Ruf AE (2015). Role of systemic inflammation in cirrhosis: from pathogenesis to prognosis. World J Hepatol.

[15] Cervoni JP, Thévenot T, Weil D (2012). C-reactive protein predicts short-term mortality in patients with cirrhosis. J Hepatol.

[16] Pearson TA, Mensah GA, Alexander RW (2003). Markers of inflammation and cardiovascular disease: application to clinical and public health practice: a statement for healthcare professionals from the Centers for Disease Control and Prevention and the American Heart Association. Circulation.

[17] Klein KB, Stafinski TD, Menon D (2013). Predicting survival after liver transplantation based on pre-transplant MELD score: a systematic review of the literature. PLoS One.

[18] Lin BY, Zhou L, Geng L (2015). High neutrophil-lymphocyte ratio indicates poor prognosis for acute-on-chronic liver failure after liver transplantation. World J Gastroenterol.

[19] Azab B, Camacho-Rivera M, Taioli E (2014). Average values and racial differences of neutrophil lymphocyte ratio among a nationally representative sample of United States subjects. PLoS One.

[20] Ramspoth T, Roehl AB, Macko S (2014). Risk factors for coagulopathy after liver resection. J Clin Anesth.

[21] (2012). Section 2: AKI definition. Kidney Int Suppl (2011).

[22] Behroozian R, Bayazidchi M, Rasooli J (2012). Systemic inflammatory response syndrome and MELD score in hospital outcome of patients with liver cirrhosis. Middle East J Dig Dis.

[23] Bonnel AR, Bunchorntavakul C, Reddy KR (2011). Immune dysfunction and infections in patients with cirrhosis. Clin Gastroenterol Hepatol.

[24] Pieri G, Agarwal B, Burroughs AK (2014). C-reactive protein and bacterial infection in cirrhosis. Ann Gastroenterol.

[25] Moreau N, Forget P, Wittebole X, Laterre P, Castanares-Zapatero D (2015). Neutrophil-to-lymphocyte ratio is associated with mortality in critically-ill cirrhotic patients. Intensive Care Med Exp.

